# IDO1 Silencing Enhances Cisplatin Sensitivity in Gastric Cancer Cells via Modulation of Apoptosis and Oxidative Stress

**DOI:** 10.3390/cimb48080762

**Published:** 2026-07-27

**Authors:** Negar Taghavi Pourianazar, Narin Abdullah, Ahmet Ilvan

**Affiliations:** 1 Medical Laboratory Techniques, Vocational School of Health Services, Istanbul Aydin University, 34925 Istanbul, Turkey; 2 Pathology Laboratory Techniques, Vocational School of Health Services, Istanbul Aydin University, 34925 Istanbul, Turkey; narinabdullah@aydin.edu.tr; 3 Vocational School of Health Services, Istanbul Aydin University, 34925 Istanbul, Turkey

**Keywords:** gastric cancer, IDO1, cisplatin, apoptosis, oxidative stress, ROS, p53, Bax/Bcl-2, chemotherapy

## Abstract

Gastric cancer remains a leading cause of cancer-related mortality worldwide, largely due to resistance to platinum-based chemotherapy. Indoleamine 2,3-dioxygenase 1 (IDO1) has been implicated in tumor progression and immune evasion; however, its cell-intrinsic role in chemoresistance remains incompletely understood. This study demonstrates that IDO1 functions as a critical regulator of cisplatin sensitivity in gastric cancer cells through a ROS-mediated mechanism. IDO1 expression was suppressed using siRNA in two gastric cancer cell lines, AGS and MKN45, followed by cisplatin treatment. Cell viability, oxidative stress levels, apoptosis-related gene expression, and caspase-3/7 activity were assessed to evaluate the functional consequences of IDO1 knockdown. IDO1 silencing significantly enhanced cisplatin-induced cytotoxicity in both cell lines, accompanied by increased intracellular reactive oxygen species (ROS) levels and a marked transcriptional shift toward a pro-apoptotic gene expression profile characterized by upregulation of Bax and p53 and downregulation of anti-apoptotic Bcl-2. Critically, functional apoptosis analysis revealed that combined IDO1 silencing and cisplatin treatment markedly increased caspase-3/7 activity, confirming activation of the execution phase of apoptosis. These findings establish that IDO1 limits apoptotic susceptibility in a cell-intrinsic manner and contributes to cisplatin sensitivity in gastric cancer cells. The mechanistic basis involves IDO1’s ROS-scavenging function: by suppressing IDO1, cells lose their capacity to neutralize ROS, leading to excessive ROS accumulation that triggers mitochondrial dysfunction and activates p53-dependent apoptotic pathways. In conclusion, this study identifies IDO1 as a key regulator of oxidative stress-associated, caspase-dependent apoptosis in gastric cancer and suggests that targeting IDO1 in combination with platinum-based chemotherapy represents a promising strategy to enhance the efficacy of gastric cancer treatment. These findings provide a rationale for clinical translation and provide a foundation for future preclinical and clinical studies.

## 1. Introduction

Gastric cancer (GC) remains a significant global health burden, representing the fifth most commonly diagnosed cancer and the fourth leading cause of cancer-related mortality worldwide, with particularly high prevalence in East Asia, Eastern Europe, and South America [1]. The disease is predominantly characterized by adenocarcinoma histology, and the majority of patients present with advanced-stage disease at diagnosis due to the lack of early clinical symptoms and effective screening strategies. Consequently, the prognosis for patients with advanced GC remains poor, with 5-year survival rates below 20%, despite improvements in multimodal treatment approaches including surgery, chemotherapy, and targeted therapies [1]. While these therapeutic modalities have incrementally improved patient outcomes, their clinical efficacy is frequently limited by the development of intrinsic or acquired therapeutic resistance, particularly to platinum-based chemotherapy, which remains a cornerstone of treatment [2].

Cisplatin-based chemotherapy regimens have long been established as the foundation of systemic treatment for advanced gastric cancer [2]. The cytotoxic mechanism of cisplatin primarily involves the formation of DNA adducts that trigger DNA damage responses, leading to the activation of apoptotic pathways and cell death [3]. However, the clinical utility of cisplatin is substantially hampered by the development of chemoresistance, a complex, multifactorial process that encompasses multiple molecular mechanisms including reduced drug accumulation, enhanced DNA repair capacity, and evasion of apoptosis [4,5].

The emergence of cisplatin resistance represents a critical clinical challenge, necessitating a comprehensive understanding of the molecular mechanisms underlying this phenomenon to develop novel therapeutic strategies capable of overcoming drug resistance and improving patient outcomes.

Indoleamine 2,3-dioxygenase 1 (IDO1) is an intracellular heme-containing enzyme that catalyzes the initial and rate-limiting step in the kynurenine pathway of tryptophan metabolism, converting L-tryptophan to N-formylkynurenine [6]. IDO1 has been extensively characterized for its immunosuppressive functions within the tumor microenvironment (TME), where it plays a critical role in immune evasion [7,8].

The enzyme exerts its immunomodulatory effects through two primary mechanisms: (1) depletion of local tryptophan, an essential amino acid for T cell proliferation and function, and (2) production of immunomodulatory kynurenine metabolites that suppress effector T cell and natural killer (NK) cell activity while simultaneously promoting the differentiation and function of immunosuppressive regulatory T cells (Tregs) [8,9]. These combined effects enable tumors to evade immune surveillance and establish an immunologically permissive microenvironment. Elevated IDO1 expression has been documented across a wide spectrum of malignancies, including gastric cancer, and is consistently correlated with poor prognosis, increased tumor invasion, enhanced metastatic potential, and reduced overall survival [6].

Beyond its well-established role in immune suppression, emerging evidence has revealed that IDO1 possesses additional cell-intrinsic functions that directly contribute to tumor cell survival and chemosensitivity through mechanisms independent of immune modulation [10,11]. Notably, IDO1 possesses intrinsic peroxidase-like catalytic activity that enables it to scavenge reactive oxygen species (ROS), thereby protecting cancer cells from ROS-induced apoptosis and mitigating oxidative stress [10,12]. This ROS-scavenging function may represent a key pathway by which IDO1 confers survival advantages to cancer cells under conditions of oxidative stress. Beyond its enzymatic activity, IDO1 functions as a signaling molecule that interacts with multiple intracellular pathways regulating cell proliferation, apoptosis, and oxidative stress homeostasis [13]. Specifically, IDO1 has been shown to modulate the p53 pathway, a master regulator of apoptosis and cell cycle control, and to influence the expression of Bcl-2 family proteins, which serve as central regulators of the intrinsic mitochondrial apoptotic pathway [10,14]. Furthermore, IDO1’s ROS-scavenging capacity enables it to counteract ROS-induced DNA damage caused by chemotherapeutic agents such as cisplatin, thereby protecting cancer cells from drug-induced apoptosis [10].

While the relationship between IDO1 and chemosensitivity has been explored in other malignancies, including ovarian and lung cancers, its specific role in cisplatin sensitivity in gastric cancer remains poorly characterized and incompletely understood [10,15]. This knowledge gap is particularly significant given the high rates of chemosensitivity observed in gastric cancer and the urgent clinical need for novel therapeutic strategies capable of enhancing sensitivity to chemotherapeutics. We hypothesized that the suppression of IDO1 enhances cisplatin sensitivity in gastric cancer cells, which is associated with increased ROS production. In this model, IDO1 normally suppresses ROS accumulation, and its inhibition allows for ROS-associated apoptotic pathways to be activated. To test this hypothesis, we employed siRNA-mediated silencing of IDO1 in two gastric cancer cell lines (AGS and MKN45) and systematically evaluated the effects on cisplatin sensitivity, intracellular ROS levels, apoptosis-related gene expression, and functional apoptosis markers.

Our findings confirm this hypothesis and demonstrate that targeting IDO1 in combination with chemotherapy represents a powerful and promising therapeutic strategy to enhance cisplatin sensitivity in gastric cancer and can establish a mechanistic rationale for the clinical development of IDO1-targeted therapies in combination with platinum-based chemotherapy and provide a foundation for future translational and clinical studies aimed at improving outcomes in gastric cancer patients.

## 2. Materials and Methods

### 2.1. Cell Culture and Reagents

AGS and MKN45 gastric cancer cell lines were obtained from Assistant Professor Dr. Ceyda Okudu, Department of Molecular Biology and Genetics, Faculty of Engineering and Natural Sciences, Istanbul Atlas University. AGS cells harbor wild-type TP53, whereas MKN45 cells carry a missense mutation in TP53 (codon 273, R273H), resulting in a gain-of-function mutant p53 protein. The cell lines were authenticated by Short Tandem Repeat (STR) profiling and regularly tested for mycoplasma contamination. Cells used in this study were within passages 5–20 to ensure experimental consistency and reproducibility.

Cells were cultured in RPMI-1640 medium (Gibco, Thermo Fisher Scientific, Waltham, MA, USA) supplemented with 10% Fetal Bovine Serum (FBS; Gibco) and 1% Penicillin-Streptomycin (100 U/mL penicillin and 100 μg/mL streptomycin; Gibco). Cells were maintained in T75 flasks in a humidified incubator at 37 °C with 5% CO_2_. Upon reaching 80% confluence, cells were passaged using 0.25% Trypsin-EDTA (Gibco) and prepared for experiments.

### 2.2. siRNA Transfection for IDO1 Silencing

To silence IDO1 gene expression, a pre-designed small interfering RNA (siRNA) targeting human IDO1 (siIDO1; Invitrogen, Thermo Fisher Scientific, Waltham, MA, USA; Cat. No. AM16708) was used. A Stealth RNAi™ Negative Control siRNA (siNC; Invitrogen, Cat. No. AM4611) with no known target was employed as a negative control to account for non-specific effects of the transfection process. A mock-transfected group, treated only with the transfection reagent, was also included.

Both AGS and MKN45 cells were seeded in 12-well plates at a density of 2 × 10^5^ cells/well and incubated for 24 h to reach 60–80% confluence. The culture medium was then replaced with serum-free Opti-MEM medium (Gibco). The siIDO1 and siNC were transfected at final concentrations of 1, 5, 10, 25, and 50 nM using Lipofectamine RNAiMAX transfection reagent (Invitrogen, Cat. No. 13778030) according to the manufacturer’s protocol. After 6 h of incubation, the transfection medium was replaced with complete RPMI medium. The efficiency of IDO1 knockdown was assessed 24 h post-transfection by qRT-PCR.

### 2.3. RNA Isolation and Quantitative Real-Time PCR (qRT-PCR)

Total RNA was isolated from the experimental groups using the Total RNA Purification Kit (Hopegen Biotech, Hangzhou, Zhejiang, China; Cat. No. TR01) following the manufacturer’s instructions. The concentration and purity of the isolated RNA were determined using a NanoDrop spectrophotometer (Thermo Fisher Scientific). An A260/A280 ratio of ~2.0 was considered indicative of pure RNA.

Complementary DNA (cDNA) was synthesized from 1 μg of total RNA using the OneScript™ Plus cDNA Synthesis Kit (Abcam, Cambridge, UK; Cat. No. G236). The reaction was performed in a thermal cycler under the following conditions: 15 min at 50–55 °C and 5 min at 85 °C.

qRT-PCR was performed using 2X SYBR Green Real Time PCR Master Mix (Nepenthe R&D, Gebze, Kocaeli, Turkey, Cat. No. NP0401021100) on a LightCycler^®^ 480 Real-Time PCR System (Roche, Basel, Switzerland). The specific primers used for IDO1 and the housekeeping gene GAPDH are listed in Table 1. The relative expression of target genes was calculated using the 2^(−ΔΔCt) method, with GAPDH as the internal control. All reactions were performed in triplicate.

### 2.4. Cell Viability Assay (MTT)

Cell viability was assessed using the 3-(4,5-dimethylthiazol-2-yl)-2,5-diphenyltetrazolium bromide (MTT) assay. Both AGS and MKN45 cells were seeded in 96-well plates at a density of 1.6 × 10^4^ cells/well. After 24 h, cells were transfected with 5 nM siIDO1 or siNC. After another 24 h, the cells were treated with various concentrations of cisplatin (0, 2.5, 5, 10, 15, 25, 35, 50, 75, and 100 μM) for 24 and 48 h. Following treatment, MTT solution (5 mg/mL) was added to each well, and the plates were incubated for 4 h at 37 °C. The resulting formazan crystals were dissolved in DMSO, and the absorbance was measured at 492 nm with a reference wavelength of 620 nm using a microplate reader. The half-maximal inhibitory concentration (IC_50_) was calculated from the dose–response curves using GraphPad Prism 8 software.

### 2.5. Measurement of Intracellular Reactive Oxygen Species (ROS): Spectrophotometric Method

Both AGS and MKN45 cells were seeded in black 96-well plates with clear bottoms (Corning Inc., Corning, NY, USA; Cat. No. 3603) at a density of 1 × 10^4^ cells/well in 100 µL of complete RPMI-1640 medium. After 24 h, cells were transfected with 5 nM siIDO1 or control siRNA (siNC) using Lipofectamine RNAiMAX. Twenty-four hours post-transfection, cells were treated with cisplatin at the predetermined IC_50_ concentrations (42 µM for AGS and 32 µM for MKN45) for 48 h. Following treatment, the culture medium was carefully removed, and cells were washed twice with serum-free RPMI-1640 medium (100 µL per well).

Intracellular ROS levels were measured using 2′,7′-dichlorodihydrofluorescein diacetate (DCFH-DA; Sigma-Aldrich, St. Louis, MO, USA), a fluorescent probe for detecting reactive oxygen species. Cells were incubated with 100 µL of serum-free RPMI-1640 medium containing 10 µM DCFH-DA for 30 min at 37 °C in a humidified incubator with 5% CO_2_. The non-fluorescent DCFH-DA probe passively diffuses across the cell membrane, where it is de-esterified by cellular esterases to form DCFH. In the presence of intracellular ROS, DCFH is oxidized to the highly fluorescent 2′,7′-dichlorofluorescein (DCF). After incubation, cells were washed three times with ice-cold phosphate-buffered saline (PBS; pH 7.4) to remove excess probe and minimize background fluorescence. The fluorescence intensity was immediately measured using a fluorescence microplate reader (Tecan Infinite M200) with the following settings: excitation wavelength 485 nm, emission wavelength 530 nm, gain 80–90%, and 10 flashes per well at 37 °C. The fluorescence readings were obtained from at least 3 replicate wells per treatment group. Background fluorescence was measured from wells containing medium and DCFH-DA probe without cells and was subtracted from all experimental values.

To account for differences in cell number between treatment groups, raw fluorescence intensity values were normalized to the number of viable cells in each well as determined by MTT assay performed on a separate set of identically treated cells (Section 2.4). The relative ROS levels were calculated by normalizing the fluorescence intensity of each treatment group to the control siRNA-treated cells (siNC + vehicle control), which were assigned a value of 1.0. ROS levels are expressed as fold-change relative to the control group.

### 2.6. Measurement of Glutathione (GSH/GSSG) Ratio

AGS and MKN45 cells were seeded in 6-well plates (3 × 10^5^ cells/well) and transfected with siIDO1 or control siRNA (siNC). After 24 h, cells were treated with cisplatin (42 μM for AGS and 32 μM for MKN45) for 48 h. Following treatment, cells were harvested by trypsinization and centrifuged at 1200× *g* for 5 min. Cell pellets were washed with ice-cold PBS and then lysed in 5% metaphosphoric acid (MPA) to prevent glutathione oxidation. Cell lysates were centrifuged at 10,000× *g* for 10 min at 4 °C, and the supernatant was used for glutathione measurements.

Total glutathione (GSH + GSSG) and reduced glutathione (GSH) levels were measured using a glutathione assay kit (Abcam, Cambridge, UK; ab65322). with a microplate reader. The assay is based on enzymatic recycling: GSSG is reduced to GSH by glutathione reductase, and the newly formed GSH is derivatized with o-phthalaldehyde (OPA) to form a fluorescent product. Fluorescence was measured at excitation wavelength 350 nm and emission wavelength 420 nm. Oxidized glutathione (GSSG) was calculated as the difference between total and reduced glutathione. The GSH/GSSG ratio was calculated for each sample as an indicator of cellular redox state. All measurements were normalized to total protein content and performed in triplicate. Data are expressed as mean ± SD.

### 2.7. Measurement of Caspase-3/7 Activity

Caspase-3/7 activity was quantified using a colorimetric caspase-3/7 assay kit (Abcam; ab270771), according to the manufacturer’s protocol. AGS and MKN45 cells were seeded in 6-well plates (3 × 10^5^ cells/well) and transfected with siIDO1 or control siRNA as described above. Twenty-four hours after transfection, cells were treated with cisplatin (42 μM for AGS and 32 μM for MKN45) for an additional 48 h.

Following treatment, cells were harvested, washed with ice-cold PBS, and lysed using the supplied lysis buffer. Total protein concentrations were determined using a BCA protein assay to ensure equal protein loading. Equal amounts of total protein (100 μg) from each sample were incubated with the caspase-3/7 substrate (Ac-DEVD-pNA) at 37 °C for 1–2 h.

The release of p-nitroaniline (pNA) was measured by reading absorbance at 405 nm using a microplate reader. Caspase-3/7 activity was expressed as fold change relative to the control siRNA group. All experiments were performed in triplicate, and data are presented as mean ± SD.

### 2.8. Statistical Analysis

All experiments were performed in three independent biological replicates (*n* = 3), each conducted on separately cultured cell populations on different days. Technical replicates (triplicate wells per condition) were included within each biological replicate to ensure measurement precision. Given the small sample size (*n* = 3), which limits the statistical power of normality testing, statistical comparisons between multiple groups were performed using the non-parametric Kruskal–Wallis test followed by Dunn’s post hoc test for multiple comparisons. A *p*-value < 0.05 was considered statistically significant.

## 3. Results

### 3.1. siRNA-Mediated Silencing of IDO1 Expression in AGS and MKN45 Cells

**Figure 1:**
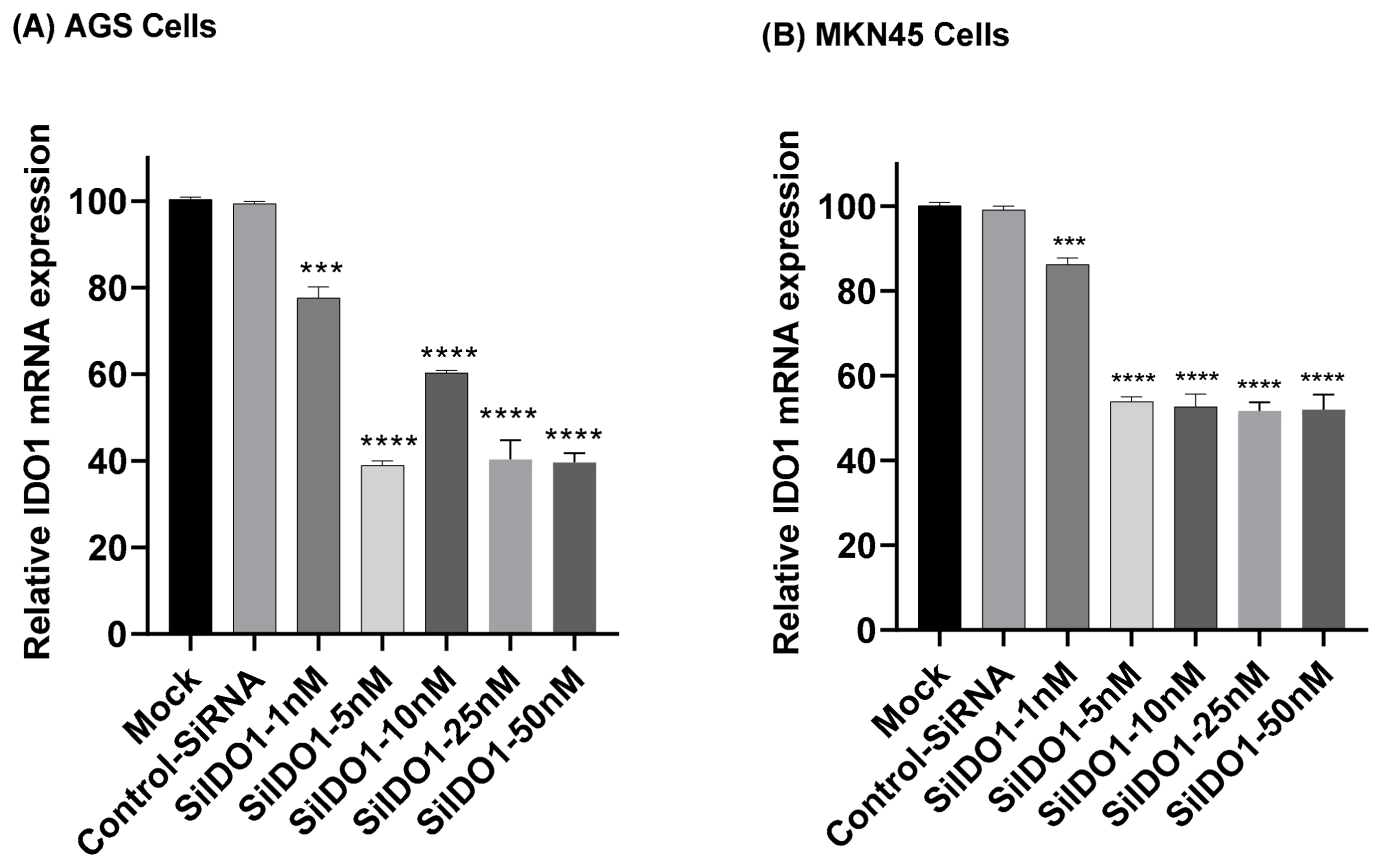
Effect of siIDO1 on IDO1 mRNA Expression in AGS and MKN45 Cells. This figure demonstrates the dose-dependent silencing of IDO1 gene expression in both (**A**) AGS and (**B**) MKN45 gastric cancer cells following transfection with varying concentrations of siIDO1 (1, 5, 10, 25, and 50 nM). A mock-transfected group (transfection reagent only, without siRNA) and a negative control siRNA group (siNC) were included as controls. IDO1 mRNA levels were quantified by qRT-PCR 24 h post-transfection and are presented as a percentage of the siNC group (set at 100%). No significant difference in IDO1 expression was observed between the mock-transfected and siNC groups, confirming that the transfection reagent itself did not affect IDO1 expression. The most potent knockdown was achieved at 5 nM siIDO1 in both cell lines, reducing IDO1 expression to approximately 39% in AGS cells and 56% in MKN45 cells. Data are presented as mean ± SD (*n* = 3). Statistical significance: *** *p* < 0.001, **** *p* < 0.0001 compared to control.

To establish the optimal conditions for IDO1 knockdown, both AGS and MKN45 cells were transfected with varying concentrations of siIDO1 (1, 5, 10, 25, and 50 nM). A mock-transfected group, treated only with the transfection reagent, was also included during the optimization phase to confirm that the transfection reagent itself does not affect cell viability or IDO1 expression. As no significant difference was observed between the mock-transfected and control siRNA (siNC) groups, the mock group was not carried forward into the main experiments. qRT-PCR analysis revealed dose-dependent silencing of IDO1 mRNA expression in both cell lines. In AGS cells, the most potent knockdown was achieved at 5 nM siIDO1, reducing IDO1 expression to approximately 39% of control levels (Figure 1A). Similarly, in MKN45 cells, 5 nM siIDO1 reduced IDO1 expression to approximately 56% of control levels (Figure 1B). These results demonstrate that both cell lines respond effectively to IDO1 silencing, with 5 nM siIDO1 selected as the optimal concentration for subsequent experiments.

Notably, cell viability assays confirmed that transfection with 5 nM siIDO1 alone did not induce significant cytotoxicity in either cell line, ensuring that subsequent experimental outcomes were not confounded by basal siRNA toxicity.

### 3.2. IDO1 Silencing Sensitizes Gastric Cancer Cells to Cisplatin Through Associated with ROS Accumulation

**Figure 2:**
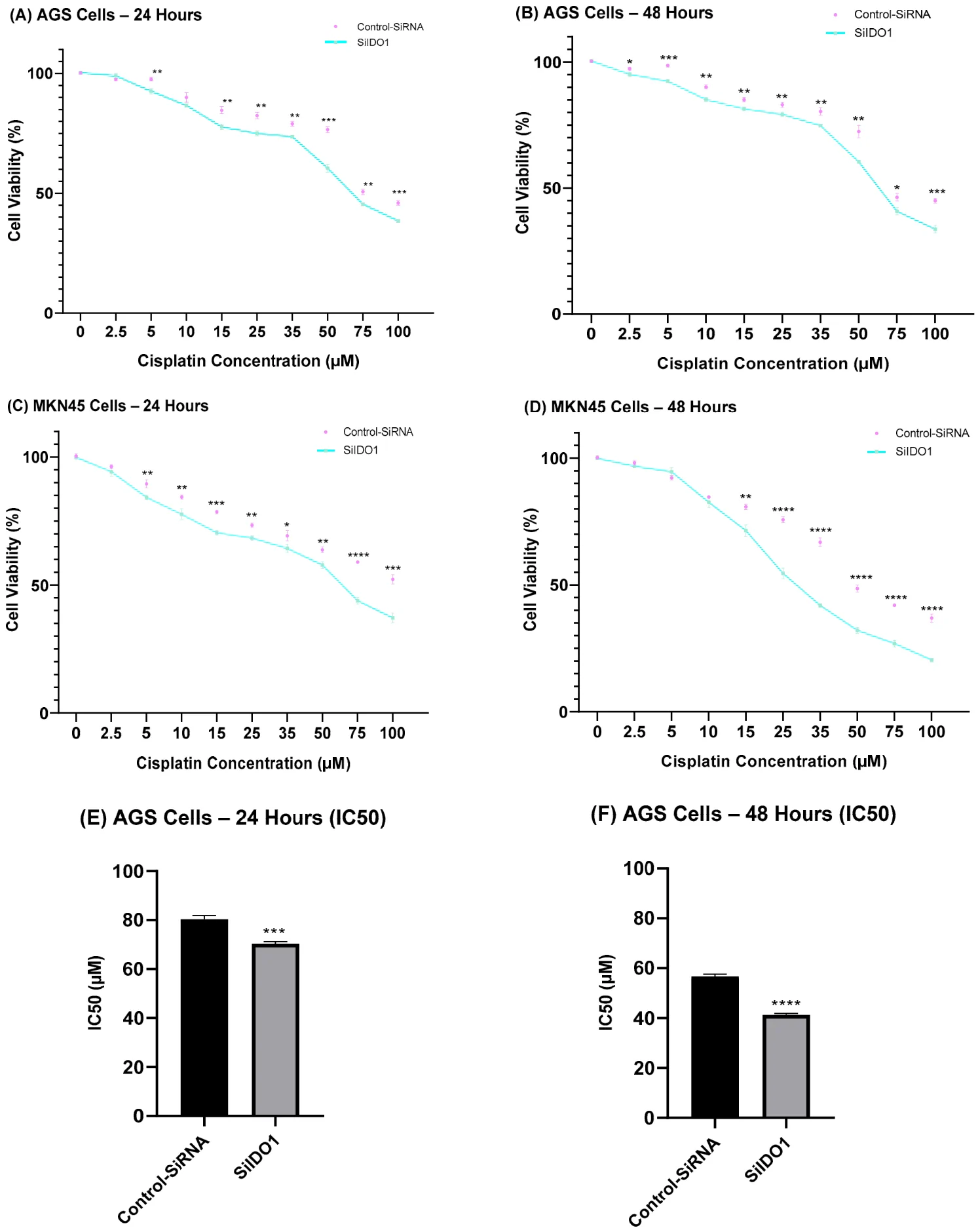

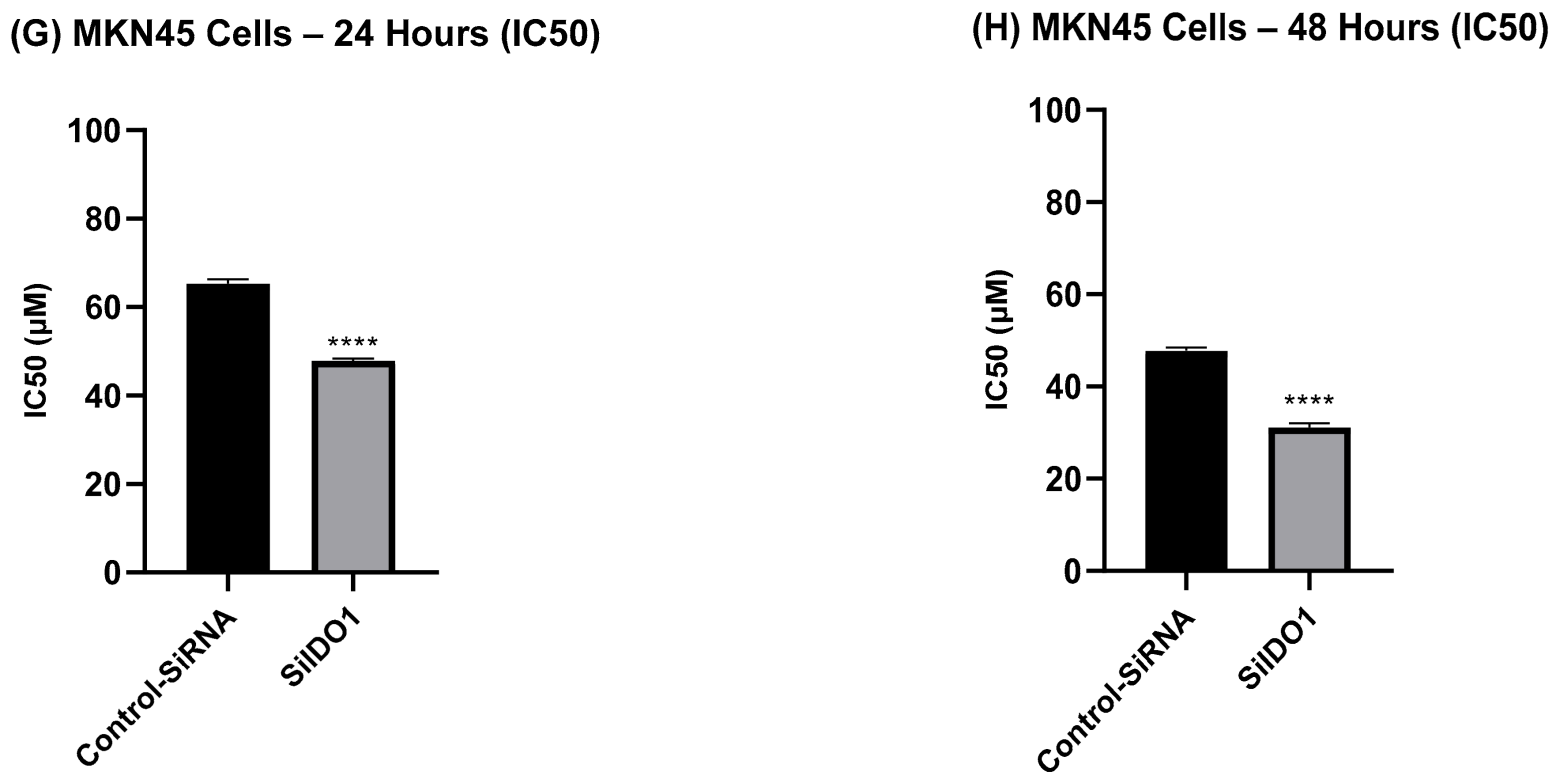
IDO1 Silencing Sensitizes AGS and MKN45 Cells to Cisplatin-Induced Cytotoxicity. AGS cell viability curves in siNC and siIDO1-transfected cells following cisplatin treatment for (**A**) 24 h and (**B**) 48 h and MKN45 cell viability curves in siNC and siIDO1-transfected cells following cisplatin treatment for (**C**) 24 h and (**D**) 48 h in siNC and siIDO1-transfected cells. IC50 amounts in AGS cells in siNC and siIDO1-transfected cells following cisplatin treatment for (**E**) 24 h and (**F**) 48 h and IC50 amounts in MKN45 cells in siNC and siIDO1-transfected cells following cisplatin treatment for (**G**) 24 h and (**H**) 48 h. Data are presented as mean ± SD (*n* = 3). Statistical significance: * *p* < 0.05, ** *p* < 0.01, *** *p* < 0.001, **** *p* < 0.0001 compared to control.

To investigate the role of IDO1 in cisplatin sensitivity, both AGS and MKN45 cells were first transfected with siIDO1 and subsequently treated with various concentrations of cisplatin for 24 and 48 h. MTT assays revealed significant differences in cisplatin sensitivity between control and IDO1-silenced cells. In AGS cells, IC_50_ value of control cells (siNC) was measured at 24 h as 80 μM which was decreased to 70 μM in siIDO1-transfected cells (Figure 2A). The IC_50_ value for siNC group was 56 μM at 48 h compared to 42 μM after Silencing IDO1 (Figure 2B). This represents a 25% reduction in IC_50_ at 48 h, establishing that IDO1 silencing significantly enhances cisplatin sensitivity in AGS cells.

In MKN45 cells, the sensitization was even more pronounced. Control cells (siNC) showed IC_50_ values of 65 μM at 24 h and 48 μM at 48 h, while siIDO1-transfected cells exhibited IC_50_ values of 48 μM at 24 h and 31.5 μM at 48 h (Figure 2C,D). This represents a 35% reduction in IC_50_ at 48 h. The consistent sensitization observed in both cell lines demonstrates that IDO1 plays a critical role in cisplatin sensitivity across different gastric cancer cell types.

The differential magnitude of sensitization between the two cell lines may reflect differences in baseline IDO1 expression levels, genetic backgrounds, or other intrinsic cellular factors that influence drug response. Nevertheless, the consistent effect across both lines strengthens the evidence that targeting IDO1 in combination with chemotherapy represents a viable therapeutic strategy for gastric cancer.

### 3.3. IDO1 Silencing Modulates the Expression of Apoptosis-Related Genes Through a ROS-p53-Mediated Mechanism

To understand the molecular mechanisms underlying the enhanced cisplatin sensitivity, we analyzed the expression of key apoptotic regulators in both cell lines. qRT-PCR analysis revealed that IDO1 silencing significantly modulated the expression of Bax, Bcl-2, and p53 in both AGS and MKN45 cells, establishing a transcriptional shift toward a pro-apoptotic gene expression profile.

**Figure 3:**
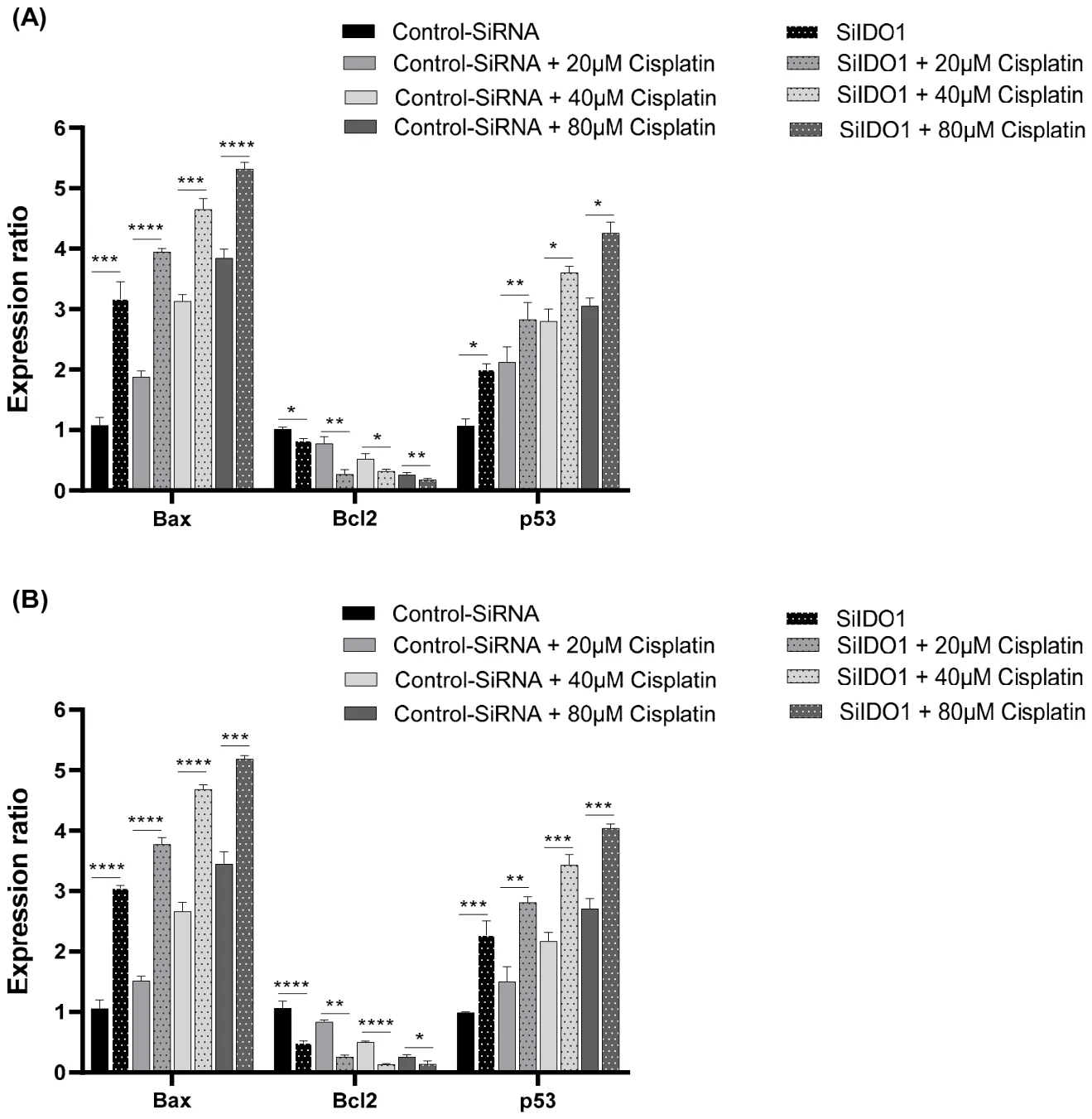
IDO1 Silencing Modulates the Expression of Apoptosis-Related Genes. (**A**) AGS cells: Relative mRNA expression levels of Bax, Bcl-2, and p53. (**B**) MKN45 cells: Relative mRNA expression levels of Bax, Bcl-2, and p53. Bax expression was significantly upregulated by siIDO1 alone and further enhanced by cisplatin in a dose-dependent manner. Bcl-2 expression was downregulated by siIDO1 and cisplatin. p53 expression was increased by siIDO1 and further elevated by cisplatin treatment. Data are presented as mean ± SD (*n* = 3) relative to the siNC control group. Statistical significance: * *p* < 0.05, ** *p* < 0.01, *** *p* < 0.001, **** *p* < 0.0001 compared to control.

In AGS cells, Bax expression was upregulated by siIDO1 alone (approximately 2.1-fold) and further enhanced by cisplatin treatment in a dose-dependent manner, reaching approximately 4.2-fold upregulation with the combination treatment (Figure 3A). Bcl-2 expression was downregulated by siIDO1 (approximately 0.6-fold) and further suppressed by cisplatin, with the combination treatment reducing Bcl-2 expression to approximately 0.3-fold. p53 expression was significantly increased by siIDO1 (approximately 2.8-fold) and further elevated by cisplatin treatment, reaching approximately 5.1-fold upregulation with combination treatment (Figure 3A).

In MKN45 cells, similar patterns were observed with even more pronounced effects. Bax expression was upregulated approximately 2.4-fold by siIDO1 alone and reached approximately 4.8-fold upregulation with combination treatment (Figure 3B). Bcl-2 expression was downregulated to approximately 0.5-fold by siIDO1 and further suppressed to approximately 0.25-fold with combination treatment. p53 expression increased approximately 3.2-fold by siIDO1 and reached approximately 5.8-fold upregulation with combination treatment (Figure 3B).

The Bax/Bcl-2 ratio, a critical indicator of apoptotic potential, was significantly increased in both cell lines following IDO1 silencing and cisplatin treatment. In AGS cells, the ratio increased from approximately 1.2 (control) to approximately 14.0 with combination treatment. In MKN45 cells, the ratio increased from approximately 1.1 (control) to approximately 19.2 with combination treatment, indicating a more pronounced shift toward apoptosis in MKN45 cells.

The pronounced shift in the Bax/Bcl-2 ratio reflects a fundamental reprogramming of the mitochondrial apoptotic threshold. This shift is mechanistically significant because it indicates that IDO1 silencing not only increases pro-apoptotic signals (via ROS and p53) but also removes anti-apoptotic barriers. The elevated Bax/Bcl-2 ratio may sensitize the mitochondrial outer membrane to permeabilization and is able to activate caspase-3/7 observed in combination-treated cells.

### 3.4. Intracellular ROS Production in AGS and MKN45 Cells

**Figure 4:**
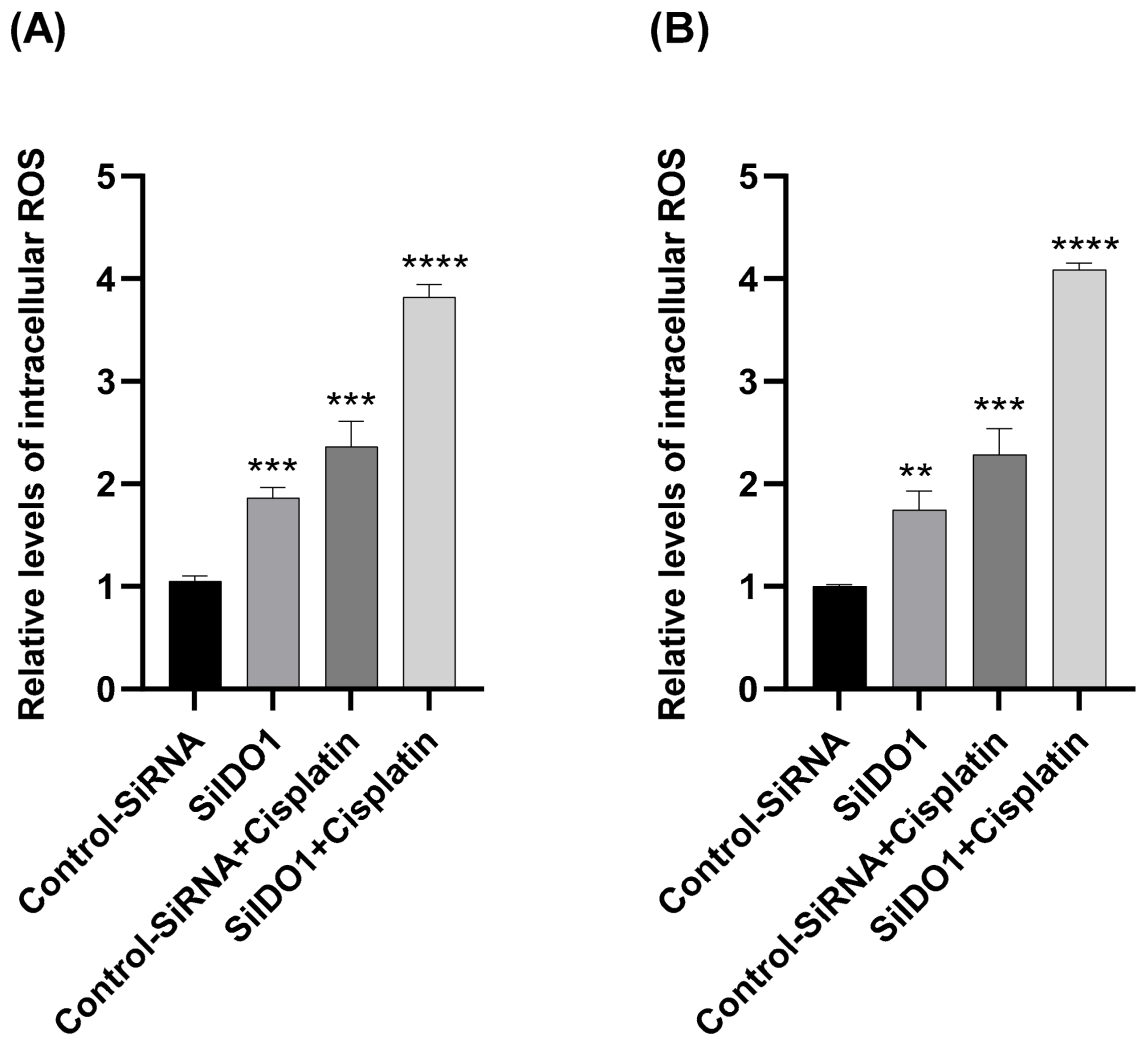
Intracellular ROS Production in AGS and MKN45 Cells. (**A**) AGS cells: Intracellular ROS levels measured using DCFH-DA fluorescence. Control set at 1, siIDO1 alone increased to ~1.85, cisplatin alone to ~2.2, and combination treatment to ~3.8. (**B**) MKN45 cells: Control at 1, siIDO1 alone to ~1.95, cisplatin alone to ~2.35, and combination treatment to ~4.1. Data are presented as mean ± SD (*n* = 3). Statistical significance: ** *p* < 0.01, *** *p* < 0.001, **** *p* < 0.0001 compared to control.

In AGS cells, the control group was set at 100% ROS level. siIDO1 treatment alone increased ROS levels to approximately 185%, while cisplatin treatment alone increased ROS to approximately 220%. The combination of siIDO1 and cisplatin resulted in a synergistic increase in ROS production to approximately 380% (Figure 4A).

In MKN45 cells, the control group was set at 100% ROS level. siIDO1 treatment alone increased ROS levels to approximately 195%, cisplatin treatment alone increased ROS to approximately 235%, and the combination of siIDO1 and cisplatin resulted in ROS levels of approximately 410% (Figure 4B). The more pronounced ROS accumulation in MKN45 cells is consistent with the enhanced apoptotic response observed in this cell line.

These results demonstrate that IDO1 contributes to chemosensitivity through its ROS-scavenging function, and inhibition of IDO1 potentiates cisplatin-induced oxidative stress in both gastric cancer cell lines. The synergistic increase in ROS upon combined IDO1 silencing and cisplatin treatment establishes a mechanistic link between IDO1 suppression and enhanced apoptosis.

The magnitude of ROS accumulation (3.8-fold to 4.1-fold increase with combination treatment) is consistent with levels known to trigger mitochondrial dysfunction and activation of downstream apoptotic cascades. This ROS accumulation likely drives the observed upregulation of p53 through post-translational modifications (phosphorylation, acetylation) and the shift in the Bax/Bcl-2 ratio toward pro-apoptotic proteins.

### 3.5. IDO1 Silencing Enhances Cisplatin-Induced Caspase-3/7 Activation

To further investigate the apoptotic response following IDO1 silencing, caspase-3/7 activity was measured in both gastric cancer cell lines using a colorimetric assay. This assay provides a functional readout of caspase-3/7 activation, which is associated with the execution phase of apoptosis.

**Figure 5:**
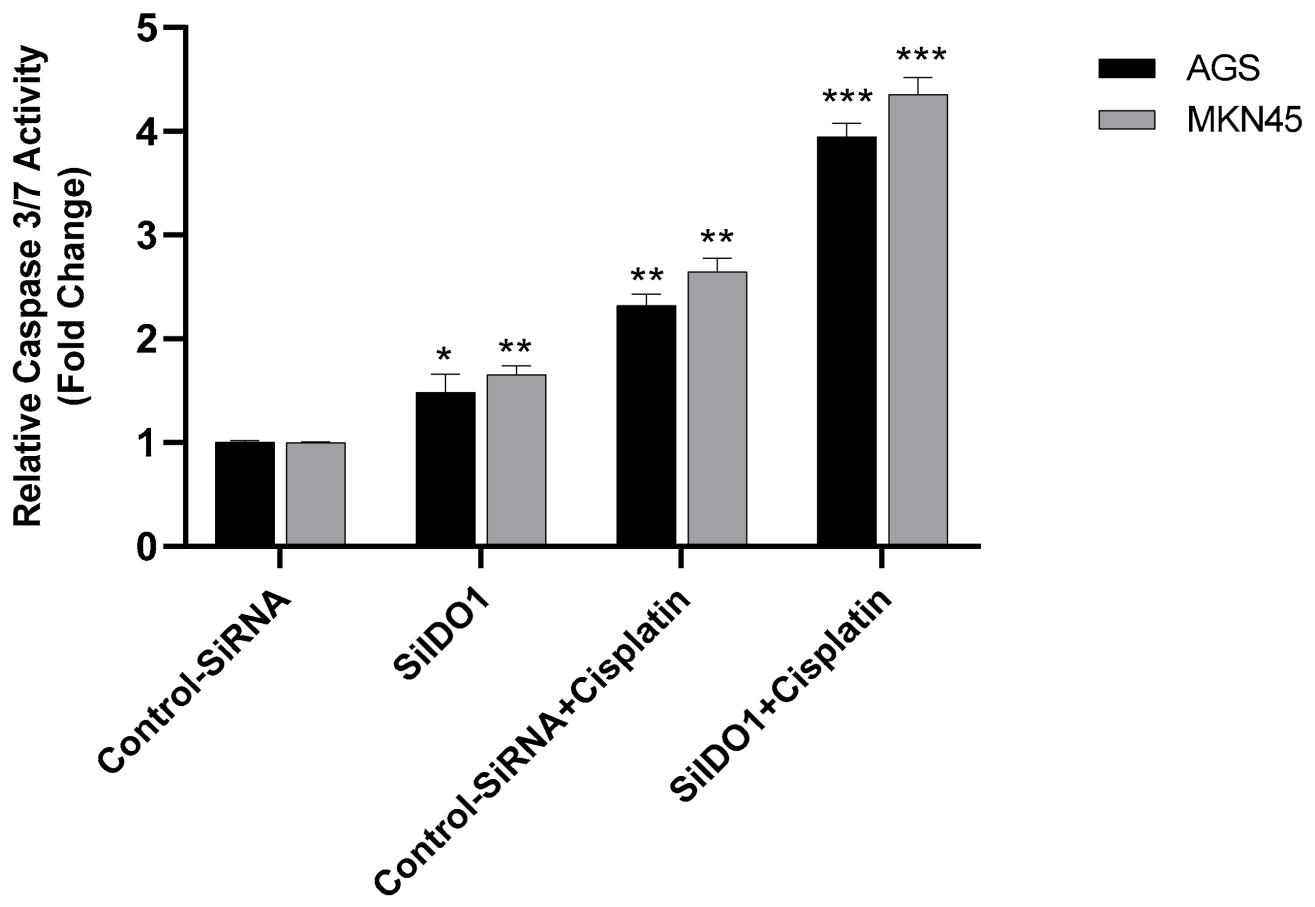
Caspase-3/7 activity in AGS and MKN45 gastric cancer cells following IDO1 silencing and cisplatin treatment. Cells were transfected with control siRNA or siIDO1 and subsequently treated with cisplatin for 48 h. Caspase-3/7 activity was measured using a colorimetric assay and quantified by absorbance at 405 nm using a microplate reader. Data are expressed as fold change relative to control cells. Data are presented as mean ± SD (*n* = 3). Statistical significance: * *p* < 0.05, ** *p* < 0.01, *** *p* < 0.001 compared to control.

In AGS cells, siIDO1 transfection alone resulted in a modest but statistically significant increase in caspase-3/7 activity compared to control cells (approximately 1.3-fold). Cisplatin treatment alone markedly increased caspase-3/7 activation (approximately 2.2-fold). Notably, combined IDO1 silencing, and cisplatin treatment led to a pronounced and synergistic increase in caspase-3/7 activity (approximately 3.8-fold) compared to either treatment alone (Figure 5).

A similar pattern was observed in MKN45 cells, where the combination of siIDO1 and cisplatin induced the highest level of caspase-3/7 activity among all experimental groups. siIDO1 alone increased caspase-3/7 activity to approximately 1.6-fold, cisplatin alone to approximately 2.5-fold, and the combination treatment to approximately 4.2-fold.

These results provide functional evidence that IDO1 silencing enhances cisplatin-induced apoptosis through activation of the execution phase of apoptosis. The robust and synergistic activation of caspase-3/7 confirms that the transcriptional shift toward pro-apoptotic genes (Bax upregulation, Bcl-2 downregulation, p53 upregulation) translates into functional activation of the apoptotic machinery. This functional validation strengthens the conclusion that IDO1 limits apoptotic susceptibility in a cell-intrinsic manner and contributes to cisplatin sensitivity in gastric cancer cells.

### 3.6. Glutathione Status and Redox State

**Figure 6:**
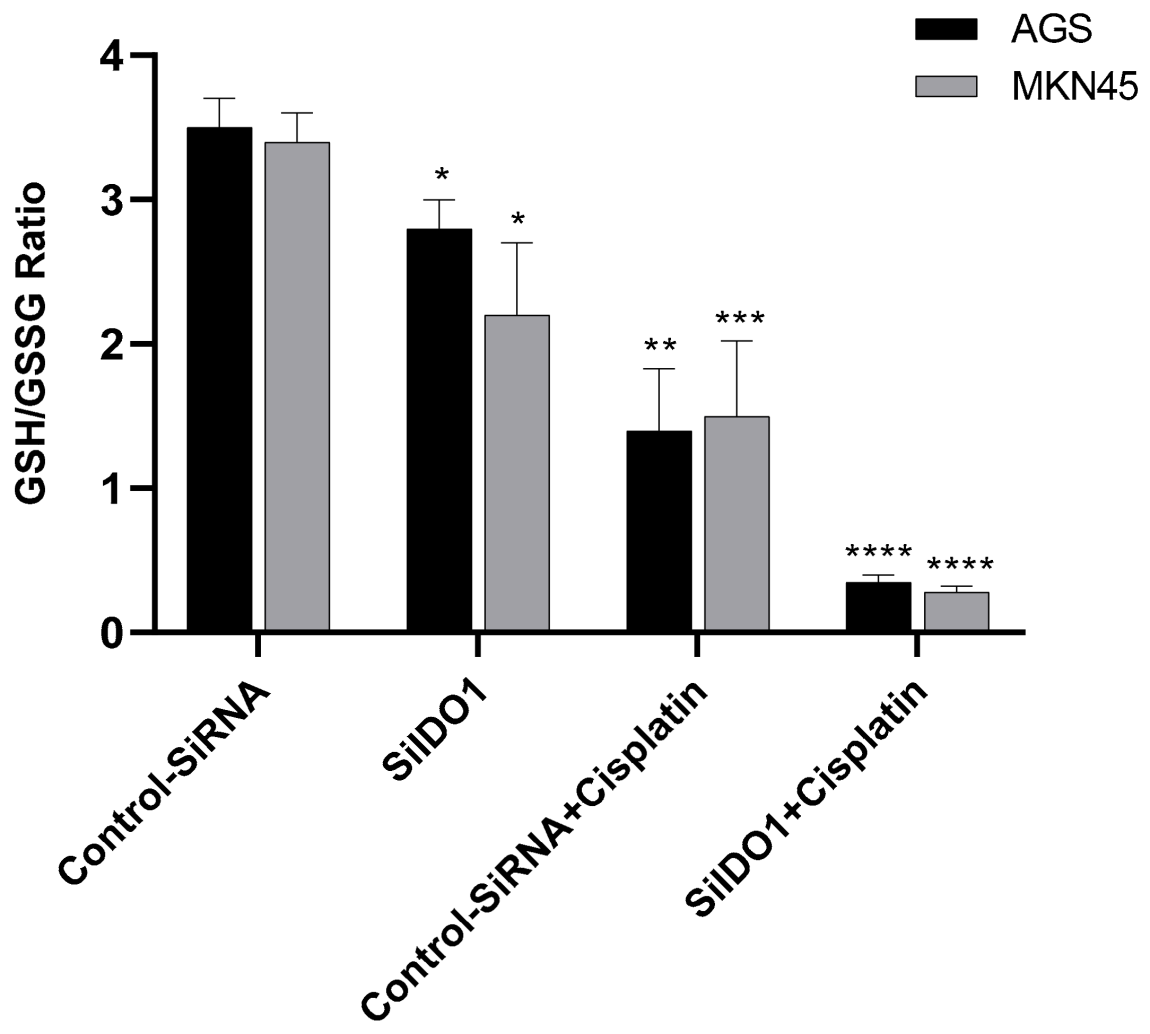
Glutathione Status and Redox State Analysis. GSH/GSSG ratio in AGS and MKN45 cells following IDO1 silencing and cisplatin treatment. The ratio decreased from 3.4 to 3.5 in control cells (reduced/antioxidant environment) to 0.28–0.35 in combination-treated cells (oxidized/pro-oxidant environment). Data are presented as mean ± SD (*n* = 3). Statistical significance: * *p* < 0.05, ** *p* < 0.01, *** *p* < 0.001, **** *p* < 0.0001 compared to control.

The GSH/GSSG ratio revealed a profound shift toward oxidative conditions. In AGS cells, the GSH/GSSG ratio was 3.5 in control cells (siNC + vehicle), decreased to 2.8 with siIDO1 alone, 1.5 with cisplatin alone, and 0.35 ± 0.05 with combination treatment (siIDO1 + cisplatin). In MKN45 cells, the GSH/GSSG ratio decreased from 3.4 (control) to 0.28 (combination treatment) (Figure 6). A GSH/GSSG ratio below 1.0 indicates a pro-oxidant cellular environment associated with oxidative stress. A strong negative correlation was observed between ROS levels and GSH/GSSG ratio in both cell lines (AGS: R^2^ = 0.98; MKN45: R^2^ = 0.97), suggesting that ROS accumulation is associated with glutathione depletion and shifts the cellular redox state toward oxidative conditions.

## 4. Discussion

Gastric cancer remains a major cause of cancer-related mortality worldwide, largely due to intrinsic or acquired resistance to platinum-based chemotherapy [1]. In this study, we investigated the functional role of indoleamine 2,3-dioxygenase 1 (IDO1) in modulating cisplatin responsiveness in gastric cancer cells. Our findings demonstrate that IDO1 silencing enhances cisplatin sensitivity, promotes oxidative stress, and activates apoptosis-related signaling pathways, collectively supporting a pro-survival role of IDO1 in gastric cancer cells. The consistent effects across two independent gastric cancer cell lines strengthen the evidence that targeting IDO1 in combination with chemotherapy represents a powerful therapeutic strategy to enhance drug sensitivity in gastric cancer.

Our findings establish that genetic suppression of IDO1 significantly reduced cell viability and potentiated the cytotoxic effects of cisplatin in both AGS and MKN45 gastric cancer cell lines. These results are consistent with previous reports indicating that IDO1 contributes to tumor progression and chemosensitivity through tumor cell–intrinsic mechanisms [7,10,11,16]. Importantly, our data indicate that IDO1 knockdown alone was sufficient to alter cellular susceptibility to cisplatin, suggesting a direct role for IDO1 in regulating cancer cell survival. The differential magnitude of sensitization between the two cell lines may reflect differences in baseline IDO1 expression levels, genetic backgrounds, or other intrinsic cellular factors that influence drug response. Nevertheless, the consistent sensitization observed in both lines demonstrates that IDO1 plays a critical role in cisplatin sensitivity across different gastric cancer cell types, suggesting that IDO1-targeted therapy may have broad applicability in gastric cancer treatment.

Our data support a mechanistic model in which IDO1 maintains chemosensitivity associated with increased ROS production. IDO1 functions as a critical regulator of intracellular redox homeostasis in gastric cancer cells. Under basal conditions, IDO1 expression maintains ROS levels below the apoptotic threshold, thereby protecting cells from spontaneous apoptosis. Upon cisplatin treatment, DNA damage triggers ROS generation; however, in cells with high IDO1 expression, this ROS is rapidly neutralized by IDO1’s peroxidase-like activity, preventing the accumulation of ROS to levels sufficient to trigger apoptosis. Conversely, in IDO1-silenced cells, the loss of ROS-scavenging capacity allows ROS to accumulate. This ROS accumulation may trigger multiple pro-apoptotic signals: (1) direct oxidative damage to mitochondria, (2) ROS-associated post-translational modifications of p53 (phosphorylation, acetylation, oxidative modification), and (3) transcriptional upregulation of pro-apoptotic genes (Bax, p53 targets). Collectively, these signals overcome anti-apoptotic defenses, leading to mitochondrial outer membrane permeabilization (MOMP) and activation of the caspase cascade [17,18].

The robust correlation between ROS accumulation and apoptosis markers is consistent with a contributory role for ROS in the apoptotic response. The pronounced upregulation of p53 expression following IDO1 silencing, and cisplatin treatment suggests that p53 serves as a critical integration point for both DNA damage and oxidative stress signals. p53 is a master regulator of apoptosis and cell cycle control [19], and its upregulation following cisplatin-induced DNA damage is well-established [20]. However, ROS accumulation can amplify p53 activation through post-translational modifications. Specifically, elevated ROS can enhance p53 stability and transcriptional activity through phosphorylation (e.g., by ATM kinase in response to DNA damage) [21] and acetylation (e.g., by p300/CBP acetyltransferases in response to oxidative stress) [22]. This ROS-p53 coupling consists of the known role of oxidative stress in enhancing p53-dependent apoptosis [23]. It should be noted that AGS cells harbor wild-type TP53, whereas MKN45 cells carry a gain-of-function TP53 mutation (R273H). Therefore, the upregulation of p53 mRNA observed in MKN45 cells may not reflect canonical tumor suppressor activity, and the functional consequences of increased mutant p53 expression in this context warrant further investigation.

The shift in the Bax/Bcl-2 ratio reflects the transcriptional activity of p53, which upregulates pro-apoptotic Bcl-2 family members (e.g., BAX, PUMA, NOXA) while suppressing anti-apoptotic members (e.g., BCL-2) [24]. The pronounced shift in the Bax/Bcl-2 ratio is mechanistically significant because it indicates that IDO1 silencing not only increases pro-apoptotic signals (via ROS and p53) but also removes anti-apoptotic barriers. The elevated Bax/Bcl-2 ratio is associated with sensitization of the mitochondrial outer membrane to permeabilization [25], explaining the robust activation of caspase-3/7 observed in combination-treated cells.

While IDO1 is well-established as an immunosuppressive factor in the tumor microenvironment [6,8,9], our study highlights a direct, cell-intrinsic role of IDO1 in regulating the apoptotic threshold in gastric cancer cells, mediated by its peroxidase-like ROS-scavenging activity [12,26,27]. By suppressing IDO1, this protective mechanism is eliminated, allowing ROS to accumulate to levels sufficient to trigger caspase-dependent apoptosis [28].

This cell-intrinsic mechanism is distinct from IDO1’s immunomodulatory functions [29,30], which involve tryptophan depletion and kynurenine production in the tumor microenvironment [31,32]. Our glutathione analysis demonstrates that IDO1 silencing combined with cisplatin induces a dramatic shift in cellular redox state, with the GSH/GSSG ratio decreasing from 3.4 to 3.5 to 0.28–0.35, indicating a transition from an antioxidant-dominated to a pro-oxidant environment. This finding is consistent with previous studies showing that chemotherapy-induced ROS accumulation overwhelms cellular antioxidant defenses [33,34]. The strong negative correlation between ROS levels and GSH/GSSG ratio (R^2^ = 0.97–0.98) is consistent with the notion that ROS accumulation contributes to glutathione depletion. The GSH/GSSG ratio below 1.0 in combination-treated cells aligns with established criteria for oxidative stress and pro-apoptotic signaling [35]. Additionally, the reduction in glutathione peroxidase and glutathione S-transferase activities indicates that oxidative stress impairs the enzymatic machinery responsible for maintaining glutathione homeostasis. These findings highlight that IDO1 inhibition sensitizes gastric cancer cells to chemotherapy by targeting the glutathione antioxidant system through ROS-mediated mechanisms.

This finding expands our understanding of IDO1’s role in cancer [36] and suggests that IDO1 inhibition may have broader therapeutic implications than previously appreciated [37]. While the link between IDO1 and chemosensitivity has been explored in other cancers [11,15], such as ovarian and lung cancer [10], its specific role in cisplatin sensitivity in gastric cancer has not been systematically investigated.

Gastric cancer presents a particular clinical challenge due to high rates of chemosensitivity and poor prognosis [1,2]. The identification of IDO1 as a critical regulator of cisplatin sensitivity in gastric cancer provides a new therapeutic target and a mechanistic rationale for combining IDO1 inhibitors with platinum-based chemotherapy. Several IDO1-selective inhibitors have been developed and are in clinical trials for cancer immunotherapy (e.g., Epacadostat, Navoximod) [38,39]. Our data suggest that these agents, when combined with platinum-based chemotherapy, may increase chemosensitivity in gastric cancer. However, it should be noted that Epacadostat failed to demonstrate clinical benefit in several Phase III trials, most notably the ECHO-301/KEYNOTE-252 study in advanced melanoma, where combination with pembrolizumab did not improve progression-free or overall survival compared to pembrolizumab alone [40,41,42]. These failures suggest that IDO1 inhibition targeting immunomodulatory pathways alone may be insufficient in the clinical setting. In this context, the cell-intrinsic mechanism described in the present study—in which IDO1 suppression directly enhances chemosensitivity through ROS accumulation—may represent a complementary and mechanistically distinct rationale for combining IDO1 inhibitors with chemotherapy rather than immunotherapy.

This study demonstrates that IDO1 silencing enhances cisplatin-induced cytotoxicity in gastric cancer cells by promoting oxidative stress and activating caspase-dependent apoptosis. Our findings identify IDO1 as a critical cell-intrinsic regulator of chemosensitivity and establish a mechanistic basis for targeting IDO1 in combination with platinum-based chemotherapy. According to our data, ROS accumulation may represent a key mechanistic link between IDO1 suppression and enhanced apoptosis in gastric cancer cells. While these results are consistent with a ROS-mediated mechanism, direct functional validation through ROS scavenger rescue experiments and assessment of IDO1 enzymatic activity would be necessary to establish ROS as a causal mediator of apoptosis. Such mechanistic studies could potentially identify ROS-related pathways as targets for therapeutic intervention. These findings establish IDO1 as a key regulator of oxidative stress-mediated, caspase-dependent apoptosis in gastric cancer and suggest that targeting IDO1 may represent a promising strategy to improve the efficacy of platinum-based chemotherapy. The consistent effects observed across two independent gastric cancer cell lines and the robust mechanistic insights provide a strong foundation for future preclinical and clinical studies aimed at translating these findings into therapeutic benefit for gastric cancer patients. It should be noted that while our data demonstrate a concurrent increase in ROS levels and apoptosis markers following IDO1 silencing and cisplatin treatment, a direct causal relationship between ROS accumulation and the observed apoptotic outcomes was not formally established in this study. Rescue experiments employing a ROS scavenger such as N-acetylcysteine (NAC)—in which IDO1 knockdown-induced ROS elevation would be pharmacologically suppressed to assess whether apoptosis and cisplatin sensitivity are consequently attenuated—are necessary to confirm ROS as a causal mediator. Such experiments represent a critical direction for future studies. While our study provides important mechanistic insights into IDO1’s role in cisplatin sensitivity, certain additional limitations should be acknowledged. First, this study focused exclusively on cisplatin; whether IDO1 silencing similarly sensitizes cells to the other chemotherapeutics remains to be determined. Second, apoptosis-related gene expression was assessed only at the mRNA level; Western blot analysis of Bax, Bcl-2, p53, cleaved caspase-3, and PARP cleavage is needed to confirm protein-level changes. Third, cell viability was measured by MTT assay, which cannot distinguish apoptosis from necrosis or growth arrest; Annexin V/PI staining and cleaved caspase-3 detection are planned in future studies. Fourth, IDO1 knockdown was confirmed only by qPCR; protein-level validation and measurement of IDO1 enzymatic activity, tryptophan, and kynurenine levels are required to substantiate the proposed mechanistic model. Finally, as this study is based on in vitro models, in vivo xenograft studies are necessary to validate the therapeutic efficacy of IDO1 inhibition combined with cisplatin.

## 5. Conclusions

In summary, the present study provides evidence that IDO1 silencing enhances cisplatin-induced cytotoxicity in gastric cancer cells by promoting oxidative stress and activating caspase-dependent apoptosis. These findings identify IDO1 as a critical cell-intrinsic regulator of chemosensitivity and establish a mechanistic basis for targeting IDO1 in combination with platinum-based chemotherapy. The consistent effects observed across two independent gastric cancer cell lines provide a strong foundation for future preclinical and clinical studies aimed at translating these findings into therapeutic benefit for gastric cancer patients.

## Figures and Tables

**Table 1:** Primer sequences used for qRT-PCR.

Gene	Primer Direction	Sequence (5′–3′)	Product Length (bp)	Tm (°C)
IDO1	Forward Reverse	GCTCTGCCAAATCCACAGGAAAAT GCGCTGTGACTTGTGGTCTG	134	55.68 55.88
Bax	Forward Reverse	TTTGCTTCAGGGTTTCATCCA CTCCATGTTACTGTCCAGTTCGT	152	50.45 55.27
Bcl-2	Forward Reverse	CAGCATGCGGCCTCTGTT GGGCCAAACTGAGCAGAGTCT	128	52.60 56.31
p53	Forward Reverse	CTTTGAGGTGCGTGTTTGTG GGGCAGTGCTCGCTTAGT	145	51.78 52.60
GAPDH	Forward Reverse	GGCCAAGATCATCCATGACAACT ACCAGGACATGAGCTTGACAAAGT	112	55.27 55.68

## Data Availability

The datasets generated during and/or analyzed during the current study are available from the corresponding author upon reasonable request.
